# Genetic variants associated with glycemic and weight loss response to vildagliptin as add-on therapy to metformin in Egyptian obese type 2 diabetic patients: potential consequences on cardiac risk factors

**DOI:** 10.1186/s40001-025-03816-5

**Published:** 2026-01-21

**Authors:** Khalid Saber, Raed Ismail, Atef Bassyouni, Mohamed El-shafee, Memy H. Hassan

**Affiliations:** 1 Department of Pharmacology and Toxicology, Faculty of Pharmacy, Al-Azhar University, Cairo, 11751 Egypt; 2 National Institute of Diabetes and Endocrinology, Cairo, Egypt; 3 Department of Clinical Pharmacy, Faculty of Pharmacy, Al-Azhar University, Cairo, 11751 Egypt

**Keywords:** Diabetes mellitus, Vildagliptin, Metformin, Genetic variations, Cardiac risk factors

## Abstract

**Background:**

This study aims to identify the genetic and clinical characteristics that affect the glycemic and weight loss responses to vildagliptin (Vilda) plus metformin (Met) among Egyptian obese type 2 diabetic (T2DM) patients. Furthermore, these responses were linked to homocysteine (Hcy) level as another cardiovascular risk factor.

**Methods:**

One hundred twenty-six obese newly diagnosed T2DM Egyptian patients (38.9% male and 61.1% female**)** fulfilling the inclusion criteria and signing the consent form were treated with Met plus Vilda for 12 months and genotyped for rs6741949 in the *DPP4*, rs6923761 in the *GLP1R*, and rs2285676 in the *KATP (KCNJ11)* genes. At 12 months post-treatment, the glycemic and weight loss-responses were defined as a reduction of the baseline HbA1c by more than 1% and a reduction of the baseline weight by more than 3% respectively.

**Results:**

The GG genotype of the *DPP4*, *GLP1R*, and *KATP* had the significant highest distribution and glycemic response to Vilda/Met than other genotypes [odds ratio (OR) = 3.55, 8.56 & 4.89; 95% confidence interval (CI) 1.13–9.51, 1.32–92.4 & 1.2–22.2; *p* = 0.033, 0.021 & 0.03, respectively]. However, the AA genotype of rs6923761 in the *GLP1R* had a significantly higher weight loss response than GA and GG genotypes (OR = 0.39 & 0.17; 95% CI 0.65–0.44 & 0.22–0.67, *p* = 0.84). Further analysis showed that lower baseline weight and BMI were associated with better glycemic and weight loss responses to Vilda/Met, while gender had no effect. Of note, glycemic and body weight loss responders show a higher reduction in Hcy level than non-responders.

**Conclusions:**

Glycemic control and body weight loss, and hence amelioration of Hcy level by Vilda as add-on therapy to Met in Egyptian T2DM obese patients are subjected to genetic variation in one or more of the *DPP-4*, *GLP1R* and *KATP* genes involved in Vilda's modes of action. The GG genotype for the three genes is associated with better glycemic response; however, the AA genotype of *GLP1R* is associated with better weight loss response. Gender has no effect; however, lower initialbody weight results in better glycemic and weight loss response.

## Introduction

Diabetes mellitus (DM) is a chronic metabolic disorder characterized by elevated blood glucose with metabolic consequences resulting from defects in insulin secretion and/or action [1]. Type 2 diabetes mellitus (T2DM) represents the predominant form of the disease, accounting for approximately 90–95% of all worldwide diabetic cases [1, 2].

Middle Eastern countries, including Egypt, are experiencing a particularly high prevalence of diabetes. The most recent International Diabetes Federation (IDF) Diabetes Atlas (2024–2025) reports that roughly 589 million adults (20–79 years) are living with diabetes worldwide. In the same line, the latest consensus concerning Egypt, the IDF estimates approximately 13.2 million adults with diabetes in 2024 (prevalence ≈ 22.4% of adults) [3].

The correlation between obesity and T2DM is well-established [4–6]. Obesity is one of the most common risk factors and has been suggested to account for 90% of the risk for T2DM with mor difficult prognosis [7]. Diabetes and obesity interplay together to induce inflammation and disturbances in lipid metabolism, which predispose to the pathogenesis of atherosclerotic cardiovascular diseases (ASCVD) [8, 9].

Uncontrolled T2DM implies micro- and macrovascular complications, especially with long duration of hyperglycemia [10, 11]. Thus, the general goal for management of T2DM is to maintain a normal blood glucose level in addition to controlling other risk factors such as obesity, oxidative stress, hyperhomocysteinemia, and dyslipidemia, which consequently overcome diabetes induced complications [12, 13].

Homocysteine (Hcy) is an amino acid whose elevated levels are linked to increased cardiovascular risk, endothelial dysfunction, and insulin resistance in T2DM. Recent evidence suggests that improved glycemic control and body weight reduction may lead to a decrease in plasma Hcy levels. Therefore, exploring the relationship between Hcy changes and pharmacogenetic response to Vilda/Met therapy may provide insights into the metabolic benefits of this combination [14, 15].

Metformin (Met) is a biguanide oral antihyperglycemic agent commonly used as the first-line medication for managing newly diagnosed obese patients [16]. Met mainly acts by increasing the effect of circulating insulin on target tissues [17].

Vildagliptin (Vilda) is an oral antidiabetic agent of the dipeptidyl peptidase-IV (DPP4) inhibitor class [18]. By inhibiting DPP4 enzyme activity, less GLP-1 is degraded, which leads to more insulin release, accompanied by inhibition of glucagon release, thus reducing fasting and postprandial hyperglycemia [19]. Although some previous studies indicated the potential cardiovascular beneficial effects of Vilda in T2DM patients, there is still no agreement about their effects on cardiovascular mortality and morbidities [20–22].

Interestingly, published clinical data and daily clinical practice have shown interindividual variability in response to Vilda among T2DM patients [23, 24]. The exact predictor of response to Vilda is not fully determined. One of the potential predictors is the genetic polymorphism of genes that mediate Vilda effects [24, 25].

A pharmacogenetic polymorphism is a common variation in a person's DNA sequence that affects how they respond to a drug. Two major types of sequence variation have been associated with variation in human phenotype: single nucleotide polymorphisms (SNPs) and insertions/deletions (indels) [23]. SNPs are an abundant form of genome variation that have a frequency of 1% or more [26]. The wild-type (or reference homozygote) genotype denotes the allele combination most frequently observed in reference populations at the locus of interest, such as “GG” for SNPs of rs6741949 in the DPP4 gene, rs6923761 in the *GLP1R* (glucagon-like peptide-1 receptor) gene, and rs2285676 in the *KATP(KCNJ11*) [ATP-sensitive potassium channel (potassium inwardly rectifying channel subfamily J member 11)] gene). A heterozygote carries one copy of the reference allele and one copy of the alternative allele “GC”, while a variant homozygote carries two copies of the same allele “CC” for (rs6741949 in the *DPP4*) or “AA” for the above-mentioned genes [26, 27].

Previous pharmacogenetic studies have reported that variations in *DPP4, GLP1R*, and *KCNJ11* genes may influence individual responses to DPP4 inhibitors, including Vilda, mainly in Asian and European populations [24, 25, 28]. However, to date, there are no published reports investigating these specific polymorphisms in relation to Vilda response among Egyptian T2DM patients. Thus, as continuation of great effort that made to identify genetic polymorphisms which predict the efficacy of antihyperglycemic drugs [20, 25, 29], this study aimed to explore genotype distribution and allelic frequencies of rs6741949 in the *DPP4* gene, rs6923761 in the *GLP1R* and rs2285676 in *KATP (KCNJ11)* gene in Egyptian obese newly diagnosed T2DM patients. In addition to identifying which genotypes of rs6741949 in the *DPP4*, rs6923761 in the *GLP1R* and rs2285676 in the *KATP* would be associated with better efficacy of Vilda/Met in providing better glycemic control and body weight reduction and hence reducing some ASCVD risk factors in Egyptian T2DM obese patients based on the crucial role of these three genes in Vilda's mode of action and previous studied in different populations [23, 30].

These potential differences could favor the pharmacogenomic selection of candidate patients to ensure better glycemic control, improved body mass index, other metabolic parameters, and hence prevention or delaying of diabetes-induced cardiovascular complications.

These three candidate polymorphisms were selected for investigation because of their mechanistic relevance to Vilda action and prior pharmacogenetic evidence. Whereas *DPP4* encodes the direct molecular target of Vilda, *GLP1R* encodes the receptor, which mediates incretin-dependent insulin secretion and KCNJ11 encodes the subunit of the ATP-sensitive potassium (KATP) channel that controls beta-cell membrane potential and insulin release. These three genes have been previously implicated in variability of insulin secretion and in pharmacogenetic responses to glucose-lowering agents. Accordingly, these three variants were considered as biological candidates for predicting therapeutic responses to Vilda [18, 24, 25, 28].

## Material and methods

### Study populations and ethical approval

This study was a prospective, single-arm, interventional study in which 136 obese Egyptian patients with newly diagnosed T2DM were initially recruited from the National Institute of Diabetes and Endocrinology, Cairo, Egypt, in the period between June 2023 and August 2024. Moreover, 10 patients discontinued treatment for unknown reasons. Consequently, 126 patients were finally enrolled in this study to receive treatment of Vilda/Met for 12 months. The patient's selection was according to the criteria of the Egyptian Ministry of Health (Egyptian Ministry of Health, 2007) and must fulfill the inclusion criteria of this study. All subjects provided written informed consent before initiation of any trial-related activities. The study was conducted in accordance with the Declaration of Helsinki and Good Clinical Practice guidelines. In addition, the protocol was approved by the Research Ethics Committee (REC), General Organization for Teaching Hospitals and Institutes (GOTHI), Cairo, Egypt. The protocol approval # IDE00302.

The inclusion criteria involve Egyptian patients with a newly documented T2DM diagnosis (naïve), 40–60 years old, HbA1c level between 6.5% (47.5 mmol/mol) and ≤ 10.0% (107.7 mmol/mol) before study initiation, BMI ≥ 30 kg/m^2^, no history of established CV disease, ability to understand and to sign a written informed consent document, outpatients, TSH within normal limit and with normal liver and kidney functions.

On the other hand, major exclusion criteria include diagnosis of T1DM, subjects with any serious medical condition requiring hospitalization, subjects with unstable cardiac disorders, renal or liver failure, subjects treated with insulin within the last 3 months, females of childbearing potential or who are pregnant, breastfeeding, subjects using any drug that could interfere with the glucose level (e.g., systemic corticosteroids) and participation in any other clinical trial.

### Study protocol

All enrolled patients were newly diagnosed obese T2DM adults who required initiation of oral antihyperglycemic therapy according to clinical guidelines. Each patient was treated with a Vilda/Met combination (Gliptus plus^®^, EVA Pharma, Egypt) in an initial daily dose of 50 mg Vilda and 500 mg Met. The medication is titrated gradually up to Vilda 50 mg and metformin 1000 mg twice daily as per the standard of care in a physician's practice [31].

The anthropometric measurements (body weight, height, and body mass index), glycemic control indicators [fasting & postprandial blood glucose (FBG & PPG) and glycosylated hemoglobin (HbA1C)], in addition to Hcy blood level, were recorded pre- and 12 months post-treatment with the Vilda/Met combination as per the standard of care in a physician's practice. Furthermore, some selected genetic polymorphism was assessed in all patients.

All patients were evaluated at each visit for any complications, body weight changes and any adverse drug reactions due to prescribed treatment. Body mass index (BMI) was calculated using the formula: BMI = kg/m^2^, where kg is a person’s weight in kilograms and m^2^ is height in meters squared. The BMI categories were classified according to WHO criteria (normal weight: 18.5–24.9 kg/m^2^; overweight: 25.0–29.9 kg/m^2^; obesity: ≥ 30.0 kg/m^2^) [32].

Based on HbA1c and body weight results, patients were classified as glycemic responders and non-responders, and weight loss responders and non-responders then related to tested genetic polymorphisms. The good glycemic response (responder) was defined as a reduction of the baseline HbA1c by > 1% [33]. While the weight responder was defined as the subject who lost > 3% of their baseline weight within 12 months [34].

#### Evaluation of glycemic response

The glycemic control in response to different treatments was evaluated by measuring serum levels of HbA1c, fasting blood glucose (FBG), and postprandial blood glucose (PPG) levels at 0 (baseline) and 12 months. Serum levels of HbA1c were determined by using HbA1c turbidimetric kits (Genesis, cat. No. 6105102, Malaga, Spain) according to the manufacturer's instructions [35]**.** However, serum levels of FPG and PPG were analyzed by a OneTouch glucometer (Life scan IP Holdings, Malvern, PA, USA) utilizing a small drop of blood taken from a fingertip [36].

#### Measurement of serum levels of homocysteine

Homocysteine was determined in serum at 0 and 12 months utilizing a Hcy ELISA kit (ELK Biotechnology, cat. No ELK8121, Wuhan, China) according to the manufacturer’s instructions [37].

#### Assessment of DDP4, GLP1R and KATP genetic variations among participants using high-resolution melting curve analysis (HRM)

Genetic variations in DPP4 (rs6741949), GLP1R (rs6923761), and KATP (KCNJ11, rs2285676) were assessed by high-resolution melting (HRM) curve analysis. The HRM assays were performed as previously described by Tanaka et al. [38]. Briefly, DNA was prepared using the QIAamp DNA Blood Mini Kit (Qiagen, Hilden, Germany) according to the manufacturer's instructions. A PCR mixture containing 10 ng template DNA and 0.7 µM forward and reverse oligonucleotide primers (final reaction volume of 25 µL) was prepared using a Type-it HRM Kit (Qiagen, Hilden, Germany). PCR amplification was performed with initial denaturing at 95 °C for 5 min, followed by 45 cycles at 95 °C for 10 s, at 52 °C for 30 s and at 72 °C for 10 s, with data acquired during the 72 °C step. For HRM analysis, amplified samples bound to the fluorescence dye were heated from 65 to 95 °C. Temperature increased by 0.1 °C at each step using Rotor-Gene Q (Qiagen, Hilden, Germany), covering the full range of the expected melting points. HRM data were analyzed using Rotor-Gene Q software. Fluorescence intensity values were normalized between 0 and 100% by defining linear baselines before and after the melting transition of each sample. The fluorescence of each acquisition was obtained by HRM curves and calculated as the percentage of fluorescence at the top and bottom baseline of each acquisition temperature, the confidence threshold being 80% [38]. PCR primers sequence and a summary of the investigated genes are listed in Table 1.

**Table 1:** Summary of primers for PCR, size and accession number of investigated genes

Gene	Forward 5′–3′	Reverse 5′–3′	Size (bp)	Accession #
*GLP-1R*	GTTCCTCTACATCATCTACAC	CTGCTTCATTCCTCTATCTG	152	AY439112.1
*KATP (KCNJ11)*	TGTCCCCTCCTAGCTGAGAC	AGGTGATGGGGAACTACCGA	178	NG_012446.1
*DPP4*	TAGCTAGCAGCACAGCACAC	TGTACAAGAAAGTTGGGTAAGGT	93	KJ896722.1

*DDP-4* dipeptidyl peptidase 4, *GLP1R* glucagon-like peptide-1 receptor, *KATP* ATP-sensitive potassium channel, *KCNJ11* potassium inwardly rectifying channel subfamily J member 11

### Statistical analysis

Only patients who completed the whole study period were included in the final statistical calculation. Mean ± SD or median and IQR were used to describe the quantitative data, while frequency was used to describe the distribution of categorical data. Normality of data was tested using Shapiro–Wilk’s test and Kolmogorov–Smirnov test. For parametric quantitative data, comparisons between two groups were made by using either a paired t-test (for the paired groups) or an unpaired t-test (for independent groups). While, the Wilcoxon sign rank test and the Mann–Whitney test were used to statistically compare nonparametric paired and unpaired data, respectively. Furthermore, categorical data were statistically compared using the Fisher’s exact test or χ^2^ test. The significance level was set at a P-value of ≤ 0.05. The sample size was precalculated using G* power V 3.1.9.4 software (Franz Faul, Universität Kiel, Germany) to detect the difference of about 0.3 between different groups with a significance criterion of α = 0.05 and power = 0.80 [39]. For genotype–response comparisons we computed odds ratios (ORs) (by logistic regression) to clarify the association between each genotype (independent variable) and the likelihood of achieving a glycemic response (dependent variable) after 1 year of treatment. The highest responsive variant was used as a reference in the statistical analysis of both glycemic response and weight loss response, while the wild type of the GG genotype was used as reference to compare variants distribution. GraphPad Prism V9.0.0 (GraphPad Prism Software Inc., San Diego, CA, USA) was used to perform statistical analysis, while the Seb Carvello online calculator was used to calculate the Hardy Weinberg Equilibrium test.

## Results

### Baseline characteristics and demographic data for the patients studied

In total, 136 obese Egyptian patients with newly diagnosed T2DM were prescribed Vilda/Met, however; 126 patients completed the experiment duration during the recruitment period between June 2023 and August 2024 (Table 2), and 10 patients discontinued treatment for unknown reasons. All patients were of Egyptian ethnicity (100%), and the mean age was 50.29 ± 8.05 years. Females were predominant, accounting for 61.11%, while males comprised 38.89%. Furthermore, the mean weight and BMI were 88.39 ± 10.4 kg and 32.83 ± 2.68 kg/m^2^ respectively. Regarding the baseline glycemic parameters, the mean HbA1c, FBG and PPG are 9.2% ± 1.44 mg/dl, 152.6 ± 2.52 mg/dl, and 258.7 ± 59.04 mg/dl, respectively. Serum Hcy level was 614 ± 86.7 (pg/ml). Overall, patients' baseline demographic, anthropometric, and glycemic characteristics ensure eligibility for subsequent enrollment in the study and treatment after signing the consent form.

**Table 2:** Baseline data for glycemic-responders and non-responders to vildagliptin/metformin treatment in enrolled Egyptian obese T2DM patients

Parameter	Responders	Non-responders	P Value	Overall
Number of patients	109 (86.5%)	17 (13.5%)		126 (100%)
Age (year)	50.45 ± 8.54	49.94 ± 8.07	0.82	50.29 ± 8.05
Males	44 (89.8%)	5 (10.2%)	0.43	49 (38.89%)
Females	65 (84.41%)	12 (15.59%)		77 (61.11%)
Height (m)	1.64 ± 0.0746	1.61 ± 0.0739	0.052	1.638 ± 0.07
Weight (kg)	87.24 ± 9.485	95.79 ± 13.03	0.0014*	88.39 ± 10.4
BMI (Kg/m^2^)	32.46 ± 2.23	35.17 ± 3.96	< 0.0001*	32.83 ± 2.682
HbA1c (%)	9.24 ± 1.47	9.15 ± 1.45	0.82	9.207 ± 1.447
PPG (mg/dl)	257.6 ± 58.42	265.8 ± 64.11	0.595	258.7 ± 59.04
FBG (mg/dl)	152.5 ± 2.46	153.3 ± 2.87	0.10	152.6 ± 2.52
Hcy (pg/ml)	613.2 ± 85.1	617.0 ± 80.78	0.863	614 ± 86.71

The glycemic responder was defined as a reduction of the baseline HbA1c by > 1% 12 months post treatment

Data are expressed as mean ± SD or frequency and percentage of the total patients or corresponding gender

The statistical analysis for parametric data was carried out using unpaired t test, while statistical analysis for non-parametric data was carried out using Fisher’s exact test

*T2DM* Type2 diabetes mellitus, *BMI* body mass index, *HbA1c* glycosylated hemoglobin, *FBG* fasting blood glucose, *PPG* post prandial glucose, *Hcy* homocysteine

P value less than 0.05 is statistically significant and indicated by *

### Glycemic response to vildagliptin plus metformin and their baseline characteristics

The good glycemic response was defined as a reduction of the baseline HbA1c by > 1% [33]. Generally, patients are sub-classified according to the glycemic response to treatment with Vilda/Met into glycemic responders and non-responders. There were 109 patients (86.5%) as glycemic responders and 17 patients (13.5%) as non-responders. The baseline characteristics of these two subgroups are presented in Table 2. The glycemic non-responder group had significantly higher baseline body weight and BMI values compared with glycemic responders (p = 0.0014 and p < 0.0001, respectively). In addition, there were no significant differences between the glycemic responders and glycemic non-responders regarding the baseline glycemic parameters (HbA1c, FBG and PPG), age, height and gender distribution (Table 2).

### The genotype distributions among the studied patients

To evaluate the influence of genetic polymorphisms on glycemic response, three single nucleotide polymorphisms (SNPs) were analyzed: rs6741949 in the *DPP4* gene, rs6923761 in the *GLP1R* gene, and rs2285676 in the *KATP* (*KCNJ11)* gene. The enrolled patients are sub-classified into 3 genotypes for each SNP. Seventy-one patients (55.5% of total) carried the GG genotype in rs6741949 of the DPP*4* gene; however, GC and CC variants were expressed in 36 patients (28.57%) and 19 patients (15.08%) respectively. Conversely, the genotypes distribution of rs6923761 in the *GLP1R* was GG in 39 patients (31%), GA in 65 patients (51.6%) and AA in 22 patients (17.4%). Similarly, the rs2285676 in the *KATP (KCNJ11)* gene was GG in 45 patients (35 0.7%), GA in 63 patients (50%), and AA in 18 patients (14.3%). Thus, the GG had the highest distribution in rs6741949 of the *DPP4* gene, while GA was the highest distributed genotype in rs6923761 of the *GLP1R* and rs2285676 of the *KATP (KCNJ11)* genes among the 126 enrolled Egyptian diabetic patients (Table 3).

**Table 3:** Distribution frequency of *DDP4, GLP1R and KATP* genotypes among enrolled Egyptian obese T2DM patients

SNP/gene	Genotypes				Overall	Hardy Weinberg
	GG	GC	CC	Non-GG		P-value
rs6741949/*DPP4*	70 (55.5%)	37 (29.4%)	19 (15.1%)	56 (44.5%)	126 (100%)	0.0008*

SNP/gene	Genotypes				Overall	
	GG	GA	AA	Non-GG		
rs6923761/*GLP1R*	39 (31%)	65 (51.6%)	22 (17.4%)	87 (69%)	126 (100%)	0.567
rs2285676/*KATP (KCNJ11)*	45 (35.7%)	63 (50%)	18 (14.3%)	81 (64.3%)	126 (100%)	0.589

The data is presented as numbers and percentages of total

Non-GG = GC + CC in *DPP4* & = GA + AA in *GLP1R* and *KATP* genes

*T2DM* Type2 diabetes mellitus, *DDP-4* dipeptidyl peptidase 4, *GLP1R* glucagon-like peptide-1 receptor, *KATP* ATP-sensitive potassium channel, *KCNJ11* potassium inwardly rectifying channel subfamily J member 11

Hardy–Weinberg equilibrium (HWE) was assessed for each SNP by χ^2^ test (df = 1) and the results are shown in Table 3. The results show no significant difference concerning rs6923761 (GLP1R) and rs2285676 (KCNJ11) (HWE: p = 0.568 and p = 0.589, respectively). Conversely, rs6741949 (DPP4) showed a significant deviation from HWE (χ^2^ = 11.16, p = 0.0008).

### Effect of genetic polymorphism on glycemic response to 1-year treatment with Vilda/Met

The genotype distributions among glycemic responders and non-responders were compared in Tables 4, 5. There was a significant association between the GG genotype in the three studied SNPs and better glycemic response in patients treated for 1 year with Vilda/Met. Where 92.8% (65 patients) of the GG genotyped patients in rs6741949 of the *DPP4* gene were significantly classified as glycemic responders compared to only five as glycemic non-responders (7.2% of total GG carrying genotype). The OR for the GG genotype was 3.55 (95% CI 1.13–9.51, p = 0.033) compared to total non-GG genotypes (Table 4). Interestingly, further analysis has shown that there was a significant difference between GG and CC genotypes (OR = 6, 95% CI 1.67–21.26, p = 0.014, Table 5). However, there was no significant statistical difference between GG and GC genotypes (OR = 2.51, 95% CI 0.65–8, p = 0.183, Table 5).

**Table 4:** Association between selected polymorphisms and glycemic response to vildagliptin/metformin treatment in Egyptian T2DM patients

SNP/gene	Genotype	Responders n = 109	Non-responders n = 17	Total n = 126	OR (95% CI)	P value
rs6741949/*DPP4*	GG	65 (92.8%)	5 (7.2%)	70 (100%)	3.55 (1.13–9.51)	0.033*
	Non-GG (GC + CC)	44 (78.58%)	12 (21.42%)	56 (100%)		
rs6923761/*GLP1R*	GG	38 (97.4%)	1 (2.6%)	39 (100%)	8.56 (1.32–92.4)	0.021*
	Non-GG (GA + AA)	71 (81.6%)	16 (18.4%)	87 (100%)		
rs2285676/*KATP (KCNJ11)*	GG	43 (95.5%)	2 (4.5%)	45 (100%)	4.89 (1.2–22.2)	0.03*
	Non-GG (GA + AA)	66 (81.5%)	15 (18.5%)	81 (100%)		

The glycemic responder was defined as a reduction of the baseline HbA1c by > 1% 12 months post treatment

The data is presented as numbers and percentages of corresponding genotypes

*T2DM* Type2 diabetes mellitus, *DDP-4* dipeptidyl peptidase 4, *GLP1R* glucagon-like peptide-1 receptor, *KATP* ATP-sensitive potassium channel, *KCNJ11* potassium inwardly rectifying channel subfamily J member 11, *OR* odd ratio, *CI* confidence interval

^*^Indicates significant difference at p ≤ 0.05 using Fisher’s exact test

**Table 5:** Association of selected genotypes with glycemic response to vildagliptin/metformin treatment in Egyptian obese T2DM patients

Variant	Responders n = 109	Non- responders n = 17	Total n = 126	OR (95% CI)	P value
rs6741949/*DPP4* gene genotypes					
GG	65 (92.8%)	5 (7.2%)	70 (100%)	Reference	
GC	31 (83.78%)	6 (16.22%)	37 (100%)	2.51 (0.65–8.0)	0.183
CC	13 (68.42%)	6 (31.58%)	19 (100%)	6.000 (1.67–21.26)	0.014*
rs6923761/*GLP1R* gene genotypes					
GG	38 (97.4%)	1 (2.6%)	39	Reference	
GA	55 (84.6%)	10 (15.4%)	65	6.91 (1.07–76.91)	0.049*
AA	16 (72.72%)	6 (27.28%)	22	14.25 (1.9–166.9)	0.007*
rs2285676/*KATP ****(****KCNJ11) gene* genotypes					
GG	43 (95.5%)	2 (4.5%)	45	Reference	
GA	52 (82.53%)	11 (17.47%)	63	4.548 (0.98–21.23)	0.0404*
AA	14 (77.77%)	4 (22.33%)	18	6.143 (1.27–33.76)	0.0299*

The glycemic responder was defined as a reduction of the baseline HbA1c by > 1% 12 months post treatment

The data is presented as numbers and percentages of corresponding genotypes

*T2DM* Type2 diabetes mellitus, *DDP-4* dipeptidyl peptidase 4, *GLP1r* glucagon-like peptide-1 receptor, *KATP* ATP-sensitive potassium channel, *KCNJ11* potassium inwardly rectifying channel subfamily J member 11, *OR* odd ratio, *CI* confidence interval

^*^Indicates significant difference at p ≤ 0.05 using Chi- square/Fisher’s exact test

A similar trend was observed in rs6923761 of the *GLP1R* and rs2285676 of the *KATP (KCNJ11)* genes*.* Whereas the GG genotype was significantly represented in 38 glycemic responders (97.4%) versus only one glycemic non-responder patient (2.6%) of rs6923761 *of* the *GLP1R*. Additionally, concerning *rs2285676 in the KATP (KCNJ11),* the GG genotype was more prevalent in glycemic responders (43 patients, 95.5%) compared to non-responders (2 patients, 4.5%). The glycemic response rate for the GG genotype in both SNPs was significantly higher than non-GG genotypes (both total and individuals as shown in detail in Tables 4 and 5, respectively).

### Changes in the anthropometric and metabolic characteristics of glycemic responders versus non-responders to 1 year treatment with Vilda/Met

The changes in glycemic control parameters (FBG & HbA1C), Hcy level, total body weight and BMI from baseline to 12 months post treatment with Vilda/Met are shown and compared in Table 6 as median and interquartile range (25–75%).

**Table 6:** Changes in anthropometric and metabolic parameters of glycemic-responders versus non-responders in Egyptian obese T2DM patients

Parameter	Glycemic response	Pretreatment (0-time)	Post treatment (12-months)	Percent change (From 0-time)	P value (Wilcoxon)
Weight (Kg)	Responders (n = 109)	87(80–95)^C^	86.2 (78.2–93)*	−1.98 (− 3.5 to −1)	< 0.0001
BMI (Kg/m^2^)		32 (30.4–33.85)^C^	31.6 (29.8–33.5)*	−1.98 (−3.5 to −1)^a^	< 0.0001
HbA1c (%)		8.7 (8.2–10.2)	7.7 (7.2–8.9)*	−11.8 (−13 to −10.3)^a^	< 0.0001
FBG (mg/dl		152.3 (150.3–154.6)	117.6 (116.4–119.1)*	−22.9 (−24.1 to −21.4)^a^	< 0.0001
Hcy (Pg/ml)		611 (526–699.8)	509 (356–721.3)*	−24.2 (−31.6–11.13)	< 0.0001
Weight (Kg)	Non responders (n = 17)	97 (89–99)	96 (87–98)*	−1 (−1 to −1)	0.0025
BMI (Kg/m^2^)		33.5 (31.6–39.3)	34.8 (31.47–38.9)*	−1 (−1 to −1)	0.0026
HbA1c (%)		9.1 (8.15–9.75)	9.3 (8.7–10.2)	3.3 (−8.8–9.5)	0.736
FBG (mg/dl)		152 (150.7–157.3)	177.5 (143.3–188)	17.3 (−6–22.45)	0.118
Hcy (Pg/ml)		611(523.5–699.8)	613 (274.8–691)	−14.3 (−50.3–8.74)	0.086

The glycemic responder was defined as a reduction of the baseline HbA1c by > 1% 12 months post treatment with vildagliptin/metformin

Data are expressed as median ± (25% −75%)

*T2DM* Type2 diabetes mellitus, *HbA1c* glycosylated hemoglobin, *FBG* fasting blood glucose, *PPG* post prandial glucose, *BMI* body mass index, *Hcy* homocysteine

^*^Indicates significant difference from corresponding zero- time at p ≤ 0.05 using Wilcoxon test

^a^Indicates significant difference from non-responder’s percent change at p < 0.05 using Mann- Whitney U test

^C^Indicates significant difference from non-responder’s zero-time value at p < 0.05 using Mann- Whitney U test

Of interest, glycemic responders had slightly non-significantly higher HbA1c values before the initiation of treatment than non-responders. However, treatment with Vilda/Met induced significant reduction in HbA1c and FBG levels in glycemic responders compared to non-responders, the median percent changes from base line values for glycemic responders were −11.6% (−13 to −10.3), p < 0.0001 & −22.8 (−24.1 to −21.24), p < 0.0001 respectively versus median positive percent changes from base line values for glycemic non responder of 3.3 (−8.8 to 9.5) and 17.3 (−6 to 22.45) in HbA1C and FBG level, respectively (Table 6).

Conversely, although glycemic responders had significantly lower initial body weight and BMI compared to non-responder group, they expressed higher reductions due to treatment compared to non-glycemic responder group (Table 6). The median percentage change of both BMI and weight from baseline values was −1.98 (−3.5 to −1) for glycemic responders compared to −1 (−1 to −1) for glycemic non-responders.

Additionally, glycemic responders showed a higher reduction in Hcy level [−24.2 (−31.6 to 11.13)] compared to the non-responders’ group [−14.3 (−50.3 to 8.74)] however, this difference did not reach statistical significance between the two groups.

Interestingly, glycemic responders showed significant reductions in weight, BMI, FBG, HbA1c and Hcy level 12 months post-treatment compared to corresponding pretreatment values. Conversely, non-responders showed a significant reduction in weight and BMI only compared to corresponding pretreatment values. However, all changes expressed by glycemic non-responders are much lower than those expressed by glycemic responders (Table 6).

### Baseline characteristics for weight loss responders versus non-responders

The weight responder was defined as the subject who lost more than 3% of their baseline weight within 12 months [34]. Accordingly, patients are sub-classified post-treatment with Vilda plus Met for 12 months into weight loss responders and non-responders. Thirty-two (25.4% of total) patients were classified as weight loss responders and 94 (74.6% of total) as weight loss non-responders. The baseline characteristics of these two subgroups are presented in Table 7. The non-responder group had significantly higher baseline BMI values compared with responders (p = 0.0065). In addition, there were no significant differences between the responders and non-responders regarding the baseline glycemic parameters (HbA1c & FBG), age, weight, height and gender distribution (Table 7).

**Table 7:** Baseline characteristics of weight-loss responders versus non-responders to vildagliptin/metformin enrolled Egyptian obese T2DM patients

Parameter	Responders (n = 32)	Non responders (n = 94)	P value
% from total	25.4%	74.6%	
Males (n = 49)	10 (20.4%)	39 (79.6%)	0.4
Females (n = 77)	22 (28.6%)	55 (71.4%)	
Age (year)	51.5 ± 8.33	50 ± 8.5	0.3887
Weight (Kg)	87.7 ± 10.99	88.63 ± 10.24	0.6621
Height (m)	1.66 ± 0.075	1.633 ± 0.066	0.0663
BMI (Kg/m^2^)	31.72 ± 1.5	33.2 ± 2.8	0.0065*
HbA1c (%)	9.1 ± 1.34	9.269 ± 1.34	0.5887
FBG (mg/dl)	152.7 ± 2.46	152.6 ± 2.56	0.95
Hcy (Pg/ml)	603.5 ± 85.86	617.3 ± 83.83	0.42

The weight responder was defined as the subject who lost > 3% of their baseline weight within 12 months

*T2DM* Type2 diabetes mellitus, *HbA1c* glycosylated hemoglobin, *FBG* fasting blood glucose, *PPG* post prandial glucose, *BMI* body mass index, *Hcy* homocysteine

Data are expressed as mean ± SD or frequency and percentage of the corresponding gender

Percentage of corresponding number

The statistical analysis for data expressed as mean ± SD was carried out using unpaired t test, while statistical analysis for data expressed as number (%) was carried out using Fisher’s exact test

Statistically significant was set at p less than 0.05

### Association between different polymorphisms and weight reduction response to Vilda/Met

The genotype distribution in relation to weight loss response for 1 year of treatment with Vilda/Met is shown in Tables 8, 9. There is no statistical difference among the wild type (GG) and non-GG variants (Table 8) or even among the three genotypes of studied SNPs for *DDP4* and *KATP* genes (Table 9). Conversely, GA and AA variants (non-GG) genotypes of the rs6923761, in the *GLP1R,* had a significantly higher weight loss response rate than homozygous patients of the wild type of GG genotype (Table 8). Further analysis showed that the AA genotype had the highest significant weight loss response among the three genotypes of rs6923761 in the *GLP1R*, where the distribution among responders and non-responders were 59.1% and 40.9% for AA versus 23.07% and 77.93% for GA and 10.25% and 89.75% for GG, respectively.

**Table 8:** Association of selected polymorphisms and weight-loss in response to vildagliptin/metformin in Egyptian obese T2DM patients

SNP/gene	Variant	Responders n = 32	Non- responders n = 94	Total n = 126	OR (95% CI)	P value
rs6741949/*DPP4*	GG	18 (25.7%)	52 (74.3%)	70 (100%)	1.04 (0.47–2.32)	0.1
	Non- GC (GC + CC)	14 (25%)	42 (75%)	56 (100%)		
rs6923761/*GLP1R*	GG	4 (10.25%)	35 (89.75%)	39 (100%)	0.24 (0.08–0.75)	0.08*
	Non- GG (GA + AA)	28 (32.2%)	59 (67.8%)	87 (100%)		
rs2285676/*KATP (KCNJ11)*	GG	9 (20%)	36 (80%)	45 (100%)	0.63 (0.27–1.44)	0.39
	Non- GG (GA + AA)	23 (28.4%)	58 (81.6%)	81 (100%)		

The weight responder was defined as the subject who lost > 3% of their baseline weight within 12 months

The data is presented as numbers and percentages of corresponding genotypes

*T2DM* Type2 diabetes mellitus, *DDP-4* dipeptidyl peptidase 4, *GLP1r* glucagon-like peptide-1 receptor, *KATP* ATP-sensitive potassium channel, *KCNJ11* potassium inwardly rectifying channel subfamily J member 11, *OR* odd ratio, *CI* confidence interval

^*^ Indicates significant difference at p ≤ 0.05 using Fisher’s exact test

**Table 9:** Association of selected genotypes with weight-loss response to vildagliptin/metformin in Egyptian obese T2DM patients

Variant	Responders n = 32	Non- responders n = 94	Total n = 126	OR (95% CI)	P value
*DPP4* genotypes					
CC	6 (31.57%)	13 (68.43%)	19	Reference	
GG	18 (25.7%)	52 (74.3%)	70	1.33 (0.42–3.76)	0.77
GC	8 (21.62%)	29 (78.38%)	37	1.67 (0.49–3.45)	0.518
*GLP1R* genotypes					
AA	13 (59.1%)	9 (40.9%)	22	Reference	
GA	15 (23.07%)	50 (76.93%)	65	0.2 (0.073–0.6)	0.003*
GG	4 (10.25%)	35 (89.75%)	39	0.08 (0.025–0.318)	< 0.0001*
*KATP* genotypes					
AA	6 (33.33%)	12 (66.37%)	18	Reference	
GA	17 (26.98%)	46 (73.02%)	63	1.35 (0.41–4.02)	0.76
GG	9 (20%)	36 (80%)	45	2 (0.56–6.3)	0.33

The weight responder was defined as the subject who lost > 3% of their baseline weight within 12 months

The data is presented as numbers and percentages of corresponding genotypes

*T2DM* Type2 diabetes mellitus, *DDP-4* dipeptidyl peptidase 4, *GLP1r* glucagon-like peptide-1 receptor, *KATP* ATP-sensitive potassium channel, *KCNJ11* potassium inwardly rectifying channel subfamily J member 11, *OR* odd ratio, *CI* confidence interval

^*^ Indicates significant difference at p ≤ 0.05 using Chi-square/Fisher’s exact test

The non-GG genotypes (CC + GC) of the rs6741949 in the *DPP4* gene had a similar weight loss response to homozygous patients of the wild-type GG genotype (Table 8). The responses were 31.57%, 25.7%, 21.62% for CC, GG and GC respectively with no statistical difference among the three genotypes (Table 9).

The AA + GA variants genotypes of rs2285676 in the KATP gene had a slightly non-significant higher weight response than homozygous patients of the wild type of GG (Table 8). The responses were 33.33%, 26.98%, 20% for AA, GA and GG respectively with no statistically significant differences among them (Table 9).

### Effect of 1 year treatment with Vilda/Met on weight and some metabolic parameters in weight loss responders versus non-responders

The anthropometric and metabolic characteristics of weight loss responders and non-responders at baseline and 12 months are shown in Table 10.

**Table 10:** Changes in measured parameters of weight-loss responders versus non-responders in Egyptian obese T2DM patients

Parameter	Weight loss	Pretreatment (0-time)	Post treatment (12-month)	% Change (From 0-time)	P value (paired design)
Weight (Kg)	Responder (n = 32)	88.5 (80–95)	79.6 (73.5–91.4)*	−3.52 (−6.34 to −3.5)^a^	< 0.0001
BMI (Kg/m^2^)		31.5 (30.3–32.9)^c^	29.3 (28.3–31.3)*	−3.52 (−6.34 to −3.5)^a^	< 0.0001
HbA1c (%)		9.4 (7.97–10.2)	8.3 (6.9–9.1)*	−11.9 (−13.27 to −10.3)	< 0.0001
FBG (mg/dl)		152.2 (150.5–155)	117.8 (116.3–119.3)*	−22.5 (−24 to −21.5)	< 0.0001
Hcy (Pg/ml)		618.5 (521–682)	374.8 (191.6–540.8)	−27.8 (−69.4 to −22.7)^a^	< 0.0001
Weight (Kg)	Non responders (n = 94)	88 (80–96)	87.6 (79.6–95.3)*	−1 (−2 to −1)	< 0.0001
BMI (Kg/m2)		32.6 (30.65–34.8)	32.4 (30.5–34.9)*	−1 (−2 to −1)	< 0.0001
HbA1c (%)		8.75 (8.2–9.82)	7.9 (7.2–9.5)*	−11.36 (−12.8 to −9.6)	< 0.0001
FBG (mg/dl)		152.4 (150.4–154.8)	118.2 (116.7–119.8)*	−22.44 (−23.7 to −21)	< 0.0001
Hcy (Pg/ml)		611 (526–716)	556 (356–778.5)*	−22.6 (−31.7 to 13.5)	0.002

The weight responder was defined as the subject who lost > 3% of their baseline weight within 12 months of treatment with vildagliptin/metformin

Data are expressed as median ± (25–75%)

*T2DM* Type2 diabetes mellitus, *HbA1c* glycosylated hemoglobin, *FBG* fasting blood glucose, *PPG* post prandial glucose, *BMI* body mass index, *Hcy* homocysteine

^*^Indicates significant difference from corresponding zero- time at p ≤ 0.05 using Wilcoxon test

^a^Indicates significant difference from non-responder’s percent change at p < 0.05 using Mann- Whitney U test

^C^Indicates significant difference from non-responder’s zero-time value at p < 0.05 using Mann- Whitney U test

Weight loss responders had significant weight reduction compared with non-responders at 12 months [median percent change from baseline is −3.52 kg (−6.34 to −3,5)] compared to median percent change of −1 kg (−2 to −1) in non-responders at 12 months. Similarly, responders had a significantly higher reduction in BMI compared with the non-responders’ group at 12 months.

Concomitantly, the weight loss responders’ group exhibited a significantly higher reduction of serum Hcy level compared to the non-responder’s group, where the median percent change from the corresponding baseline values were −27.8 (−69.4 to −22.7) and −22.6 (−31.7 to 13.5) for responders and non-responders, respectively.

Conversely, there was no significant statistical difference between the percentage change from baseline of glycemic control parameters (HbA1c and FBG) in weight loss responders versus non-responders (Table 10).

Both weight loss responders and non-responders showed a significant reduction in weight, BMI, HbA1c and FBG post-treatment compared to pretreatment, while non-responders only showed a significant reduction in Hcy post-treatment compared to the corresponding pretreatment value (Table 10). However, the magnitudes for reductions for all parameters are much higher in weight loss responders than in non-responders.

Of note, responders experienced significantly lower BMI values at baseline before the initiation of Vilda/Met treatment as shown in Tables 7, 10.

## Discussion

This prospective study was carried out in the period between June 2023 and August 2024. The study included 126 Egyptian obese newly diagnosed T2DM patients who completed 1 year of therapy with the Vilda/Met combination. The principal aim was to identify common genetic genotypes in DPP4 (rs6741949), GLP1R (rs6923761), and KCNJ11/KATP (rs2285676) that are associated with improved glycemic control and body-weight response. These genetic associations may contribute to better protection against diabetes-induced cardiovascular complications. The protective effect is likely mediated through the modulation of major risk factors, including obesity, hyperglycemia, and hyperhomocysteinemia. Understanding these genetic influences may help optimize treatment efficiency while minimizing adverse effects and reducing overall therapy costs.

Overall, the enrolled patients exhibited similar baseline demographic, anthropometric and glycemic characteristics, taking into consideration both the inclusion and exclusion criteria which ensure the comparability for subsequent analyses of treatment efficacy.

This study confirmed that Vilda as add-on therapy to Met significantly improved glycemic control, as indicated by reductions in HbA1c by more than 1% of the baseline value in 86.5% of enrolled patients (glycemic responders), in addition to a significant reduction in FBG [40, 41]. However, it should be noted that 13.5% of the enrolled patients are glycemic non-responders. Additionally, our continuing research data and some earlier data showed that the lower cardiovascular benefits of DDP4 inhibitors, including Vilda, than dapagliflozin as add-on therapy to Met [22, 42], hence, to unravel this weakness, it was pertinent to explore the potential effect of genotype difference and gender differences of enrolled patients on glycemic response and weight loss response to Vilda plus Met as CVS risk factors and furthermore, explore the potential role of glycemic control and weight loss on Hcy level as another CVS risk factor [9, 13, 14, 43].

Interestingly, our study documented and confirmed the previously published results that the treatment was more effective in glycemic control among certain genotypes of genes responsible for the mode of action for antidiabetic drugs, including DPP4 inhibitors, than other genotypes [23–25, 29, 30]. A novel aspect of our study was the assessment of genetic variations for SNPs rs6741949 in *DPP4*, rs6923761 in *GLP1R*, and rs2285676 in *KATP (KCNJ11)* and their impact on drug efficacy. These three genes were selected since they mediate Vilda’s hypoglycemic effect [18]. We found that *DPP*4 (rs6741949), *GLP1R* (rs6923761) and *KATP* (rs2285676) polymorphisms significantly affected the glycemic response to Vilda/Met however, only *GLP1R* (rs6923761) affected the weight loss response to Vilda/Met.

Subgroup analysis showed that the non-glycemic responders had significantly higher baseline body weight and BMI values compared with responders although there were no significant differences between the glycemic responders and non-responders regarding the baseline glycemic parameters (HbA1c, FBG and PPG), age, height and gender. These findings indicate that higher baseline weight and BMI might be associated with poor glycemic response to Vilda/Met. In addition, age and gender distribution did not significantly influence glycemic response. These results are in line and confirm previous studies reporting similar conclusions [6, 7, 9, 21, 43, 44].

Additionally, our results document that the wild type of GG genotype in all studied SNPs of the three genes (*DPP4*, *GLP1R* and *KTAP*) was significantly associated with a higher rate of glycemic response to Vilda/Met combination, suggesting a potential pharmacogenetic influence on Vilda efficacy. However, variants GC and CC genotypes of the rs6741949 in the *DPP4* gene, GA and AA genotypes of both the rs6923761 in the *GLP1R* and the rs2285676 in the *KATP* gene had a lower glycemic response than the wild-type (GG) genotype indicating lower treatment efficiency. These findings highlight the importance of genetic screening in personalizing diabetes treatment, particularly for patients receiving DPP-4 inhibitors such as Vilda. These results are in line and furthermore, solidify previously published results [23–25, 29, 30, 33].

Moreover, our results indicated that the wild type of GG has the highest distribution in the *DDP4* gene among the participant patients, while the GA variant has the highest distribution in the *GLP1R* and *KATP* genes which might be the cause behind the reported efficacy of Vilda/Met among the studied population (86.5%) since the main mechanism for Vilda is mediated via DDP4 inhibition [18].

However, the lower efficacy in glycemic non-responders (13.5%) to Vilda/Met therapy that had been reported in this study on Egyptian obese diabetic patients and in previously published articles on other ethnic groups that reported lower efficacy compared to SGLT 2 inhibitors such as dapagliflozin alone or in combination with Met [22, 42, 45] could be attributed to the genetic differences in *GLP1R* and or *KATP* genes. Where the GG genotype, which has the highest hypoglycemic efficacy, had a lower distribution among enrolled patients than the GA + AA genotypes of rs6923761, in the *GLP1R* and rs2285676, in the *KATP* gene.

It’s worth mentioning that weight loss and BMI reduction were significantly higher in glycemic responders than glycemic non-responders although they had significantly lower initial weight and BMI than glycemic non-responders, which might confer additional cardiovascular benefits beyond glycemic control against risk factors [9, 13, 43]. Noteworthy, these results highlight the importance of weight management strategies when prescribing DPP-4 inhibitors, particularly for obese patients. This is consistent with previous findings that obese T2DM patients have more insulin resistance and require additional interventions to achieve optimal glycemic control [6, 7, 9, 43, 44, 46, 47].

Hyperhomocysteinemia is one of the major cardiovascular risk factors contributing to atherosclerosis [14, 15]. Our results reported that only glycemic responders exhibited a significant reduction in Hcy level post-treatment compared to pre-treatment, which was not achieved in the glycemic non -responders’ group. These results highlighted the importance of good glycemic control to protect against hyperhomocysteinemia and hence potential cardiovascular diseases and in turn highlight the importance of genetic screening in personalizing diabetes treatment to achieve the best cardiovascular protection.

Although we observed a higher trend toward greater reductions in plasma Hcy among glycemic responders compared to non-responders; however, the percentage change between the two groups did not reach statistical significance. Several factors may explain this finding such as low sample size specially in non- responder group, interindividual variability in Hcy concentrations that has been reflected by wide interquartile range in non- responder group, the potential influence of multiple metabolic factors in Hcy level such as folate/vitamin B12 status and Met dose among studied patients. Future studies with larger sample sizes, with controlled folate/vitamin B12 status are warranted to determine whether the observed trend reflects a true metabolic effect of improved glycemia or is attributable to confounding and measurement variability [48, 49].

Concerning weight loss response, overall results of all enrolled (126) patients demonstrated that Vilda/Met treatment for 12 months induced weight loss by more than 3% of the corresponding baseline weight value in only 25.4% of enrolled patients. Previous studies indicated that DDP4 inhibitors, including Vilda, are body weight neutral [41]. However, other studies reported that Vilda may induce body weight gain with good glycemic control [42]. However, some studies have reported modest weight loss effects with Vilda [13, 50]. Furthermore, meta-analysis found that Vilda improves glycemic control and may have additional benefits on cardiovascular outcomes, including weight reduction, especially when combined with Met [51, 52]. These controversial results may be due to different ethnicities of the studied population, dose, patients’ conditions, treatment duration or study design. The controversy has also extended to the effect of Met on body weight [16, 53].

Based on our results herein, and to unravel previous researchers’ controversial results concerning the effect of Vilda/Met on diabetic patients’ weight, especially obesity, is one of the crucial risk factors for cardiovascular health [9, 13, 43], we expanded our study to explore the effect of genetic polymorphism on weight reduction response to Vilda/Met combination, utilizing the same SNPs utilized in glycemic response as mentioned above especially our enrolled patients were initially obese.

Body weight loss response subgroup analysis showed that the baseline characteristics for both weight loss responders and non-responders to the Vilda/Met have no differences in all measured parameters, including gender, age, initial weight and HbA1c. However, like glycemic responders, the weight loss responder group had a significantly lower initial BMI than the non-responder group, which again addressed the importance of initial BMI control along therapy with DDP4 inhibitors to achieve better treatment efficacy in T2DM [54, 55].

Interestingly, the highest weight loss response rate was observed in non-GG genotypes, especially the AA genotype of *GLP1R;* however, there was no significant difference between GG and non -GG in other genes studied, indicating the differential genetic difference between weight loss response and glycemic response to Vilda/Met. Unfortunately, patients carrying the AA genotype in *GLP1R* gene constitute 17.4% only of total enrolled subjects, which could be the reason for the lower weight loss response rate reported in our study and, in part, unravel the lower body weight loss response to Vilda in previous studies [13, 41, 42, 50].

The results are consistent with previously published articles that reported that genetic differences in *GLP1R* affect incretin-based therapies, including DPP-4 inhibitors [29, 56]. Furthermore, the rs6923761 gene variant of GLP1R is involved in weight loss, glycemic control, and cardiovascular risk, especially the AA variant [30, 57]. However, previously published articles indicated that pharmacogenomic effects can be population-specific [23, 30], suggesting that further research in Egyptian cohorts versus non-Egyptians is necessary to confirm and generalize the conclusion.

Worth mentioning that the percent reduction in levels of Hcy from baseline was significantly higher in weight loss responders than weight loss non-responders and even it was better than glycemic responders, which reconfirms and points out the importance of weight reduction and its association with better glycemic control to ameliorate cardiovascular risk factors including obesity, hyperglycemia and hyperhomocysteinemia [9, 13–15, 43, 47].

Thus, selecting patients with certain genetic polymorphisms could confer better glycemic response and or weight loss response to Vilda/Met. Furthermore, good glycemic control and better weight loss could in turn, confer better control on Hcy level and eventually provide better cardiovascular protection against diabetic induced CVS risk.

No significant differences were observed between males and females in terms of baseline glycemic parameters or response to therapy concerning glycemic response and weight loss response, which could omit the effect of gender in Vilda/Met efficacy on blood glucose and body weight. However, a larger sample size may be needed to confirm whether hormonal or metabolic factors influence drug efficacy between both sexes.

Although our study has succeeded in fulfilling its aim and being to our knowledge, the first article dealing with pharmacogenetic variability in response to Vilda/Met and exploring the distribution of genetic genotypes in the three genes in the Egyptian population. The study linked the variability in weight loss and glycemic control to Hcy level as a cardiovascular risk factor. In addition, this study has many other strength points such as being randomized, powered, a prospective interventional study, non-biased by genetic heterogeneity of patients (all recruited patients are Egyptians from the same locality), all patients were monitored in the same institution. We believe our results entail a relevant significance in the prediction of good glycemic control, weight loss control in obese newly diagnosed T2DM Egyptian patients, and hence CVS risk control such as high Hcy serum level in addition to hyperglycemia and obesity.

Another merit is that we analyzed polymorphisms using both codominant and dominant genetic models to comprehensively explore how each genotype affects therapeutic response patterns. This approach was selected to increase statistical power and simplify interpretation of the genetic effect on analyzed outcome. Using both models allows detection of different inheritance patterns and is consistent with standard published articles reporting pharmacogenetic variability and their outcome in treatment response [30, 33]. The reported results showed associations of GG genotype with highest rate of glycemic response under both models, confirming robustness of the genetic effect on treatment efficacy.

The major limitation of our study is the relatively small number of participants and relatively short duration to confirm the cardiovascular benefits. Future research should focus on the cardiovascular benefits of Vilda/Met in responder patients with different ethnicities, utilizing large numbers of participants to confirm the associations.

## Conclusion

The Vilda/Met combination for 1 year is successful in reducing HbA1c in Egyptian obese newly diagnosed T2DM patients by more than 1% in 86.5% of enrolled patients. Additionally, this combination has also reduced the total body weight by more than 3% of initial body weight (weight loss responder) in 25.4% of total enrolled patients. These responses are subjected to genetic variations; however, gender has no effect. Glycemic responders and body weight loss responders show a higher reduction in plasma Hcy level than non-responders.

The GG variant, highly distributed in the *DPP4* rs6741949, *GLP1R* rs6923761, and *KATP* rs2285676 genes among Egyptian obese diabetic patients, was significantly associated with a higher rate of glycemic response compared to other tested genotypes, indicating a pharmacogenetic influence on Vilda/Met efficacy. The *GLP1R* rs6923761 variant AA showed the highest rate of weight loss response to Vilda/Met compared to GA and AA genotypes. However, the tested genotypes of *DPP4* rs6741949 and *KATP* rs2285676 have no significant statistical difference in weight loss response. Indicating the pivotal role of pharmacogenetic variability in *GLP1R* for weight loss response. Because overall responder probability is high in our cohort, ORs for non-response can appear large.

Thus, the assessment of genetic variations in *DPP4* rs6741949, *GLP1R* rs6923761, and *KATP* rs2285676 at the baseline is a good predictor of glycemic response before applying the therapy with Vilda/Met in Egyptian T2DM patients. However, *GLP1R* rs6923761 is a good predictor of both glycemic and weight loss responses. Both a good glycemic response and better weight loss response are associated with a significant reduction in Hcy serum level, which might protect against ASCVDs. Moreover, this suggests treatment continuation (GG) or discontinuation (non-GG), especially in the presence of severe treatment-related side effects, suboptimal patient motivation or severe comorbidities.

## Data Availability

The datasets supporting the conclusions of this article are included within the article. Any further details about the data and materials of this study are transparent and available upon reasonable request.
